# The Possible Role of the Nitroso-Sulfide Signaling Pathway in the Vasomotoric Effect of Garlic Juice

**DOI:** 10.3390/molecules25030590

**Published:** 2020-01-29

**Authors:** Andrea Berenyiova, Marian Grman, Anton Misak, Samuel Golas, Justina Cuchorova, Sona Cacanyiova

**Affiliations:** 1 Institute of Normal and Pathological Physiology, Centre of Experimental Medicine, Slovak Academy of Sciences,841 04 Bratislava, Slovakia; samuel.golas@savba.sk (S.G.); sona.cacanyiova@savba.sk (S.C.); 2 Institute for Clinical and Translational Research, Biomedical Research Center, Slovak Academy of Sciences, 845 05 Bratislava, Slovakia; marian.grman@savba.sk (M.G.); anton.misak@savba.sk (A.M.); 3 Axxence Slovakia, Ltd., 811 07 Bratislava, Slovakia; justina.cuchorova@axxence.sk

**Keywords:** garlic, vasoactive mechanisms, NO donor, H_2_S donor

## Abstract

The beneficial cardiovascular effects of garlic have been reported in numerous studies. The major bioactive properties of garlic are related to organic sulfides. This study aimed to investigate whether garlic juice works exclusively due to its sulfur compounds or rather via the formation of new products of the nitroso-sulfide signaling pathway. Changes in isometric tension were measured on the precontracted aortic rings of adult normotensive Wistar rats. We evaluated NO-donor (S-nitrosoglutathione, GSNO)-induced vasorelaxation and compare it with effects of hydrogen sulfide (H_2_S)/GSNO and garlic/GSNO. Incubation with garlic juice increased the maximal GSNO-induced relaxation and markedly changed the character of the relaxant response. Although incubation with an H_2_S donor enhanced the maximal vasorelaxant response of GSNO, neither the absolute nor the relative relaxation changed over time. The mixture of GSNO with an H_2_S donor evoked a response similar to GSNO-induced relaxation after incubation with garlic juice. This relaxation of the H_2_S and GSNO mixture was soluble guanylyl cyclase (sGC) dependent, partially reduced by HNO scavenger and it was adenosine triphosphate-sensitive potassium channels (K_ATP_) independent. In this study, we demonstrate for the first time the suggestion that H_2_S itself is probably not the crucial bioactive compound of garlic juice but rather potentiates the production of new signaling molecules during the GSNO-H_2_S interaction.

## 1. Introduction

The beneficial biological effects of garlic have been well known for a long time; garlic is a commonly used ingredient of traditional medicine worldwide. Recent studies have shown that the bioactive components of garlic provide many biological functions. The bioactive components of garlic are effective antioxidants, immunomodulators and anti-inflammatory, cardiovascular protective, anticancer, hepatoprotective, antidiabetic, anti-obesity, neuroprotective, renal protective, antibacterial and antifungal agents [1,2,3,4,5,6]. Moreover, it has also been demonstrated that the intake of garlic powder can effectively reduce blood pressure, total cholesterol, low-density lipoprotein cholesterol, and other risk factors of cardiovascular diseases [7]. The major bioactive properties of garlic are related to organic sulfides, such as allicin, alliin, diallyl sulfide, diallyl disulfide, diallyl trisulfide, ajoene and S-allyl-cysteine [8,9]. These bioactive molecules are able to decrease proliferation and migration of the vascular smooth muscle cells, inhibit angiotensin converting enzyme, increase nitric oxide (NO) bioavailability and increase hydrogen sulfide (H_2_S) production so they can decrease vasoconstriction and enhance vasorelaxation in the arterial bed [10].

Gaseous signal molecules, such as NO and H_2_S, are endogenously generated by several enzymes and have been demonstrated as influencing a wide range of physiological and pathological processes [11]. Both molecules have been shown to induce several cytoprotective mechanisms that include induction of vasodilatation, inhibition of apoptosis, modulation of mitochondrial respiration, activation of antioxidants, promotion of angiogenesis and inhibition of inflammation [12].

H_2_S and H_2_S-derived compounds induce potent biological effects due to binding to a metal center of heme proteins or because of post-translational modification of cysteine residues called protein persulfidation (S-sulfhydration); by its interaction with protein disulfides or protein sulfenic acids, H_2_S can be transported within the organism and released at the target location [13,14]. Similarly, low-molecular-weight thiols bind and transport another important gaseous transmitter, NO [15]. The cross-talk between H_2_S and NO has also been intensively studied [16,17,18,19,20,21]. The chemical interaction between H_2_S and NO is primarily based on the production of nitrosothiols, such as S-nitrosoglutathione (GSNO) [22]. It has been demonstrated that H_2_S (HS^−^) releases NO from GSNO, potentiating vascular smooth muscle relaxation [16]. Moreover, several other studies have suggested the existence of an interaction between NO/H_2_S carriers resulting in the formation of new products [17,18,19,20]. These new signaling molecules would trigger quantitatively different vasoactive effects than NO and H_2_S alone due to their qualitatively different mechanisms [21]. Recently they are considered to be a part of a new and original nitroso-sulfide signaling pathway. Products of this H_2_S/NO interaction appear to have pronounced specific biological effects. The nitroxyl (HNO) is a potential candidate, but several other compounds have been suggested to mediate the bioactivity of the interaction between S-nitrosothiols and H_2_S. For example, thionitrous acid (HSNO) has been proposed [18]. Cortese-Krott et al. [20] observed that sulfide reacted with S-nitrosothiols to form multiple bioactive products and proposed that nitrosopersulfide (SSNO^-^) could account for some of the longer-lived effects of the interaction between S-nitrosothiols and H_2_S. However, the nature of the reaction intermediates is still subject of scientific studies.

Recent study has confirmed that garlic extract also interacts with endogenous NO, affects NO production and exerts endothelium-dependent vasorelaxation of the aorta in normotensive rats [23]. This study aims to investigate whether garlic juice or an exogenous H_2_S donor affects the GSNO-induced vasorelaxation. Another aim of this study is to determine whether garlic extract works exclusively due to its sulfur compounds or rather via the formation of new products related to the nitroso-sulfide signaling pathway.

## 2. Results

### 2.1. Gas Chromatography Mass Spectrometric (GC-MS) Analysis of the Garlic Juice

**Figure 1:**
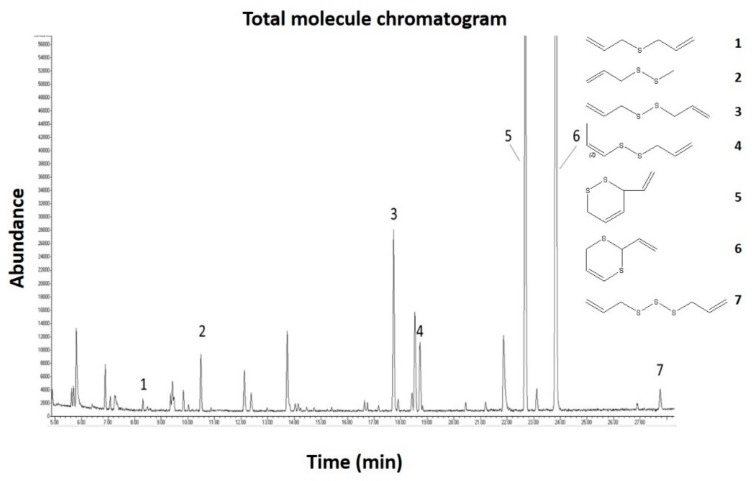
The total molecule chromatogram from gas chromatography mass spectrometric (GC-MS) analysis of the garlic juice. The exact organo-sulfur compounds could be identified and their formula are shown: **1** diallyl sulfide, **2** 3-(Methyldisulfanyl)-prop-1-ene, **3** diallyl disulfide, **4** (*Z*)-1-(prop-2-enyldisulfanyl)prop-1-en, **5** 3-ethenyl-3,6- dihydrodithiine, **6** 2-ethenyl-4*H*-1,3-dithiine and **7** diallyl trisulfide.

GC-MS analysis confirmed the presence of several organic sulfur compounds in the garlic juice. (Figure 1). The analysis identified seven major sulfur-containing molecules in the samples. The total molecule chromatogram is showing the peaks for each compound. The samples of garlic juice contained diallyl sulfide, diallyl disulfide and diallyl trisulfide, which are considered to be active compounds with high bioactive properties (see Introduction). 

### 2.2. Effect of Garlic Juice Incubation

**Figure 2:**
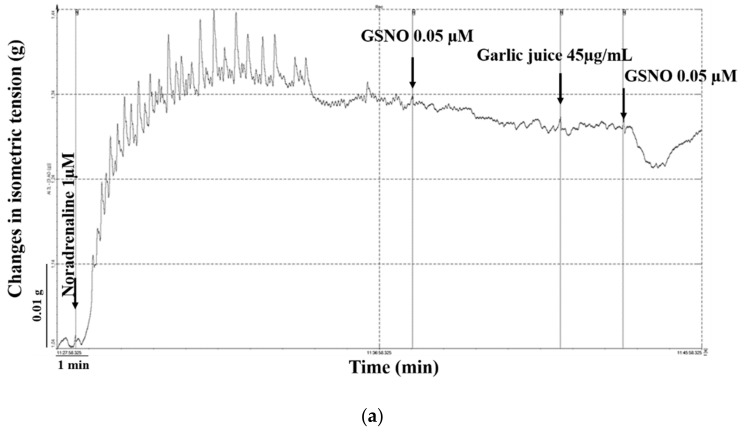

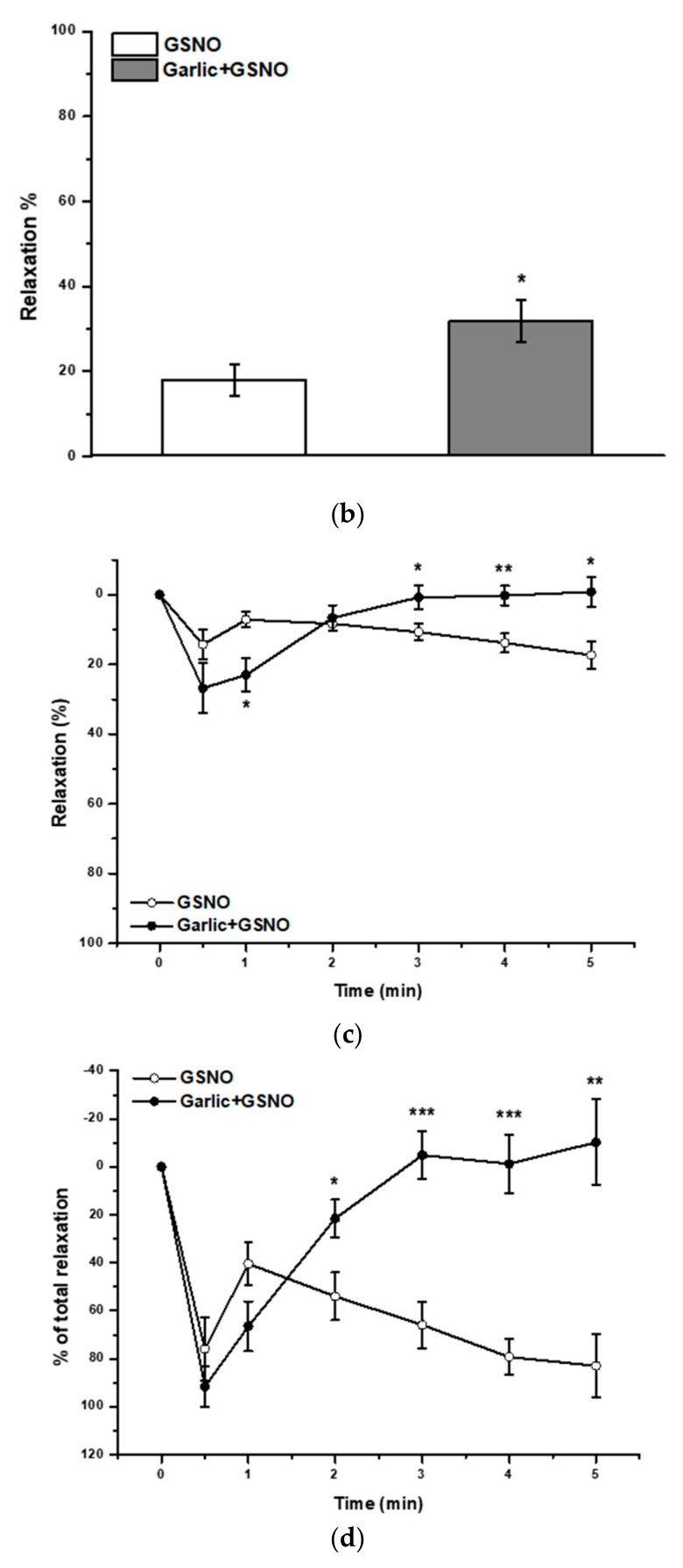
The comparison of the vasorelaxation induced by S-nitrosoglutathione (GSNO) alone and after incubation with garlic juice. Original record of the incubation with garlic juice (**a**). The comparison of the maximal relaxation (**b**) and the absolute (**c**) as well as the relative (**d**) relaxation in time induced by GSNO (0.05 µM) alone and by GSNO (0.05 µM) after incubation of precontracted aortic rings with garlic juice (45 ug/mL) (*n* = 9). Values are mean ± S.E.M. * *p* < 0.05; ** *p* < 0.01; *** *p* < 0.001 with respect to the value of the GSNO response.

A three-minute long incubation with garlic juice (45 ug/mL; *n* = 9) did not affect the vascular tension of the noradrenaline-precontracted aortic rings (1 µM) (Figure 2a), however it significantly increased (*p* < 0.05) the maximal GSNO-induced relaxation (Figure 2b). The incubation with the garlic juices markedly changed the character of the GSNO-induced relaxant response. The absolute relaxation was significantly increased at the 1^st^ minute (*p* < 0.05) but reduced at the 3^rd^, 4^th^ and 5^th^ min (*p* < 0.05; *p* < 0.01; Figure 2c). The expression of the relative relaxation over time showed a faster return after incubation with the garlic juice (*p* < 0.05; *p* < 0.01; *p* < 0.001; Figure 2d). 

### 2.3. Effect of H_2_S-Donor Incubation

**Figure 3:**
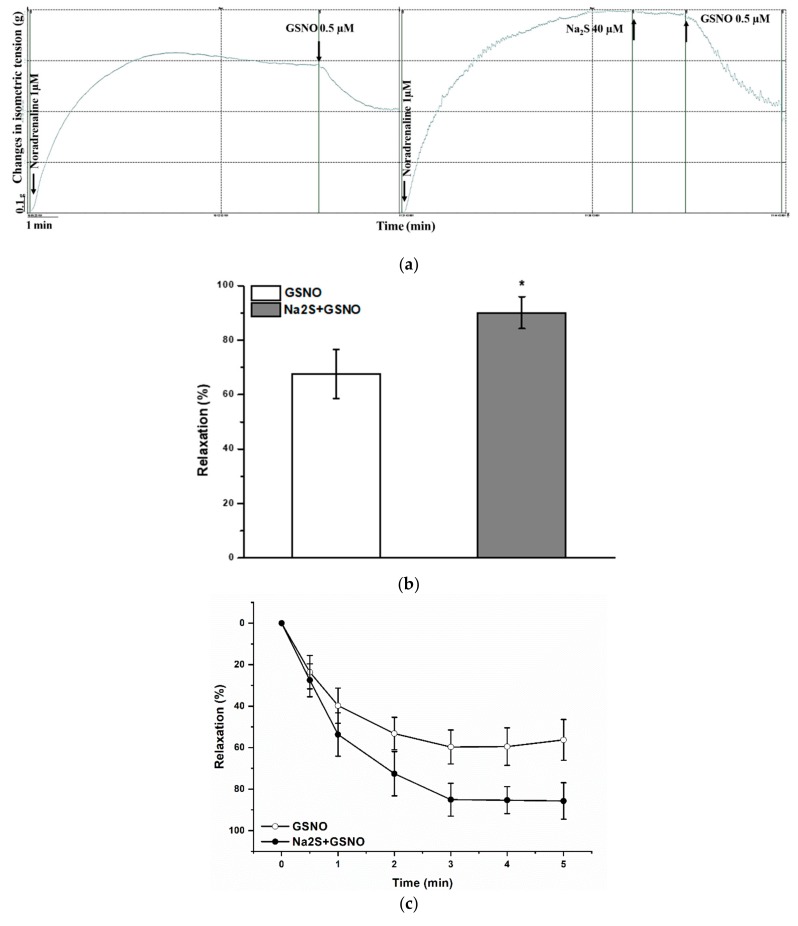

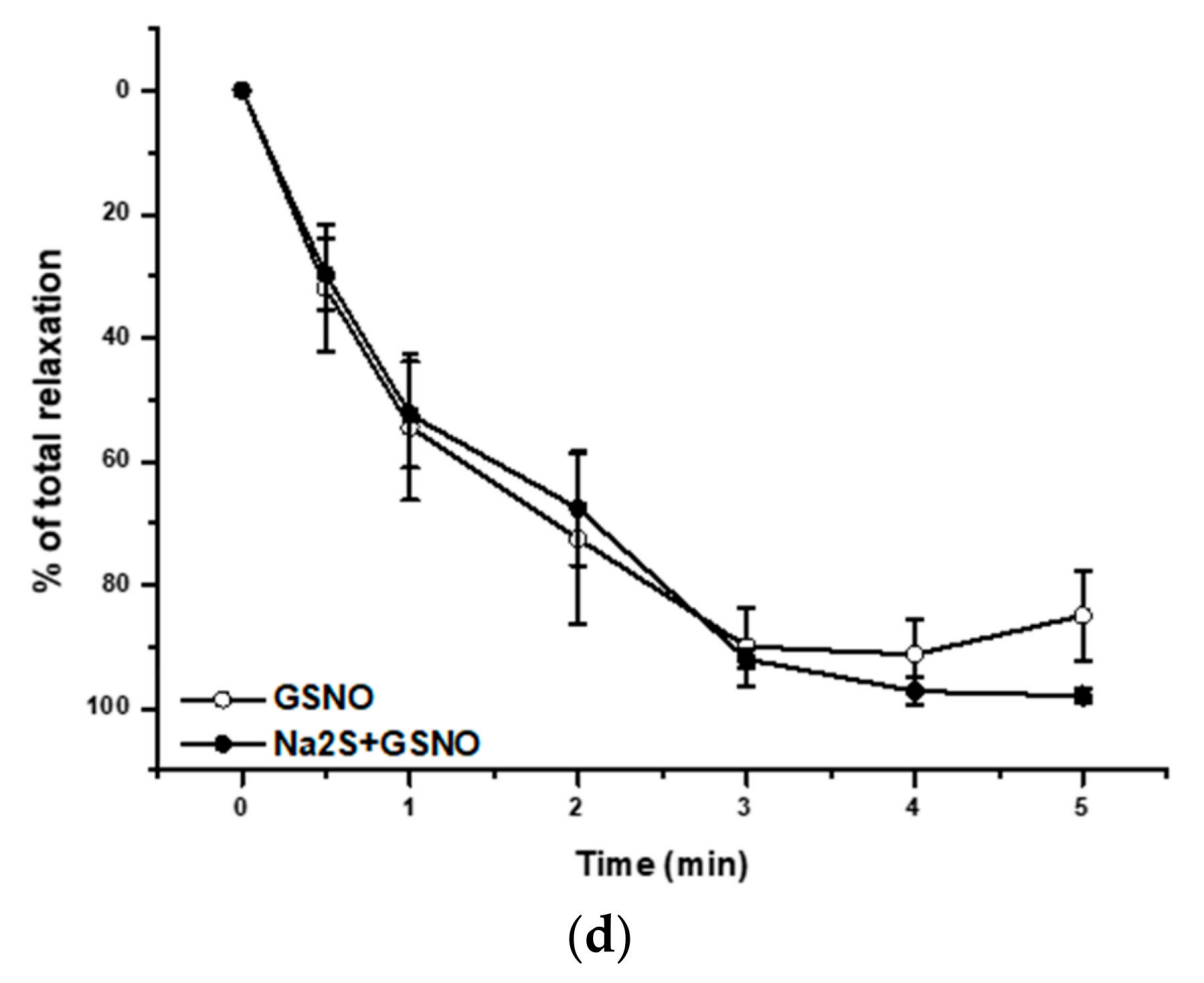
The comparison of the vasorelaxation induced by GSNO alone and after incubation with Na_2_S. Original record of the incubation with Na_2_S (40 µM) (**a**). The comparison of the maximal relaxation (**b**) and the absolute (**c**) as well as the relative (**d**) relaxation in time induced by GSNO (0.5 µM) alone and by GSNO (0.5 µM) after incubation of the precontracted aortic rings with Na_2_S (40 µM) (*n* = 8). Values are mean ± S.E.M. * *p* < 0.05.

We incubated the noradrenaline-precontracted aortic rings with the H_2_S donor, Na_2_S (40 µM; *n* = 8) for 3 min. This concentration of Na_2_S did not have a significant effect on the arterial tone (Figure 3a). However, it modified the maximal vasorelaxant response of precontracted aortic rings to GSNO (0.5 µM; *n* = 8). The maximum enriched relaxation was significantly increased after incubation with Na_2_S compared with the relaxation induced by GSNO alone (*p* < 0.05; Figure 3b). The absolute relaxation over time was not changed (Figure 3c). Moreover, incubation with Na_2_S did not affect the speed of the relaxation (Figure 3d).

### 2.4. Effect of the H_2_S/GSNO Products

**Figure 4:**
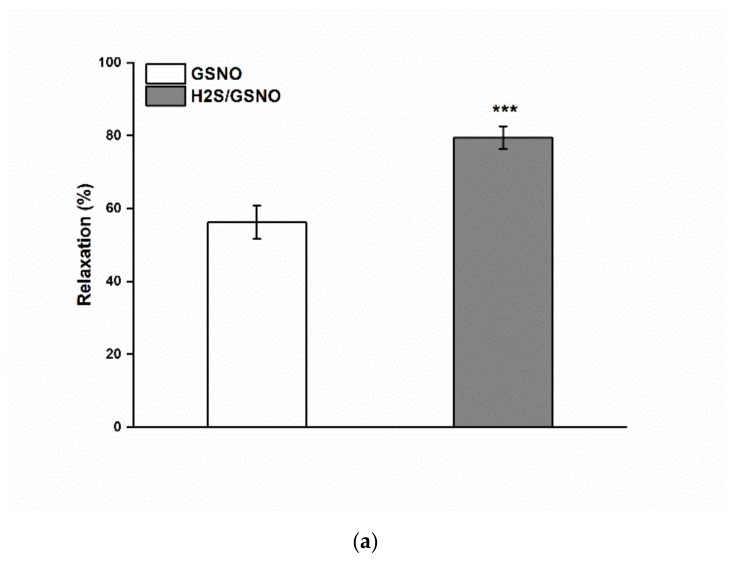

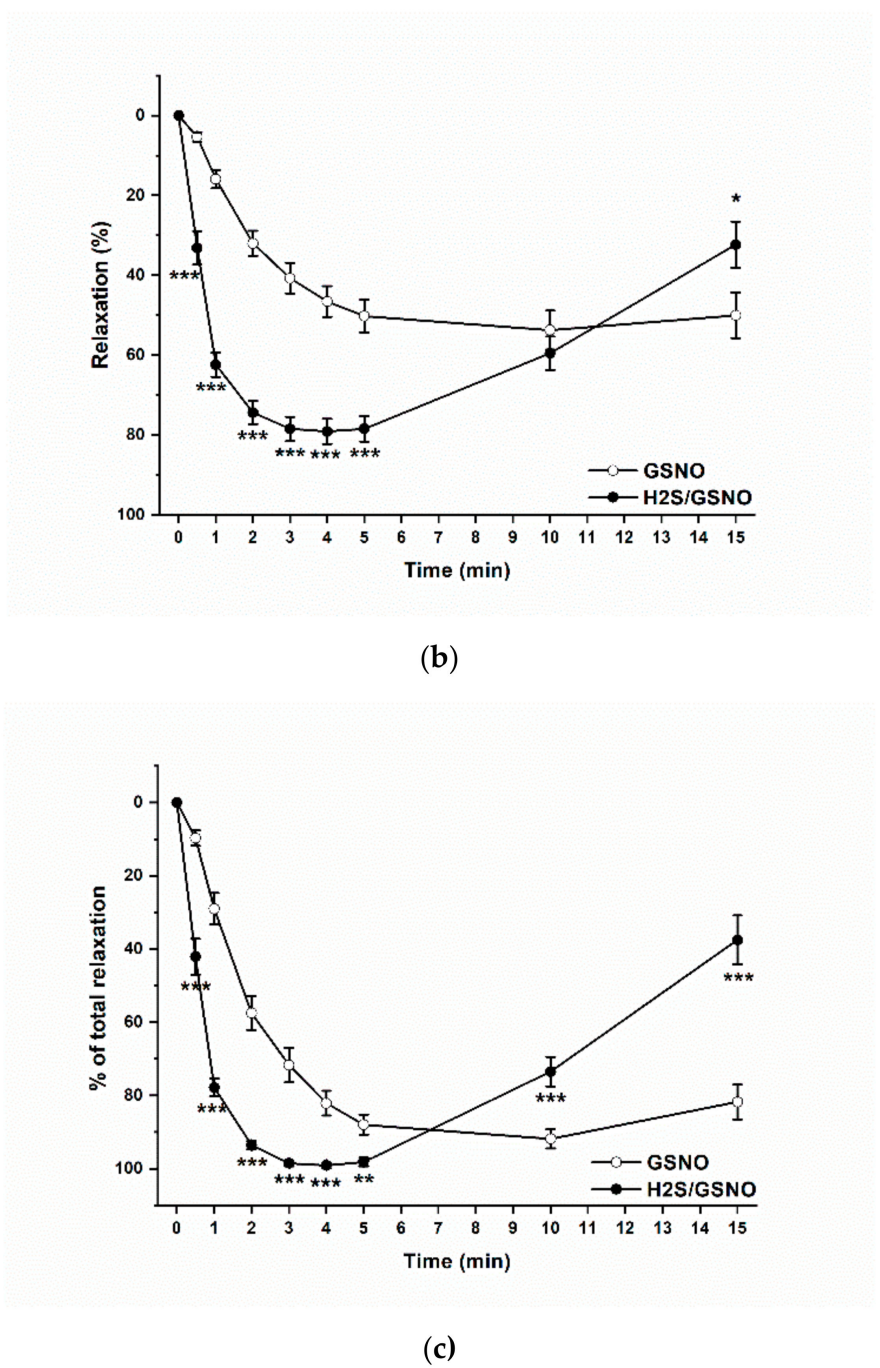
The comparison of the vasorelaxation induced by GSNO alone and by H_2_S/GSNO products. The comparison of the maximal relaxation (**a**) and the absolute (**b**) as well as the relative (**c**) relaxation in time induced by GSNO (0.5 µM) alone and by H_2_S/GSNO products (0.5 µM) (*n* = 19). Values are mean ± S.E.M. * *p* < 0.05; ** *p* < 0.01; *** *p* < 0.001 with respect to the value of the GSNO response.

The H_2_S/GSNO products (0.5 µM: mixture of 5 µM Na_2_S + 0.5 µM GSNO; *n* = 19) induced a significantly enhanced maximal vasorelaxation compared to GSNO alone (*p* < 0.001; Figure 4a; *n* = 19). The H_2_S/GSNO products (0.5 µM) triggered a pronounced absolute vasorelaxation (at the 0.5^th^, 1^st^, 2^nd^, 3rd, 4^th^ and 5^th^ min), followed by a quick return (at the 15^th^ minute) (*p* < 0.05; *p* < 0.01; *p* < 0.001; Figure 4b). The time-dependent relative relaxation was also different at all the measured points: the initiation of relaxation (0.5^th^, 1^st^ and 2^nd^ minute), achievement of maximal relaxation (3^rd^, 4^th^ and 5^th^ minute) and return (10^th^ and 15^th^ minute) were faster after H_2_S/GSNO product application than those after GSNO application alone (*p* < 0.01; *p* < 0.001; Figure 4c).

**Figure 5:**
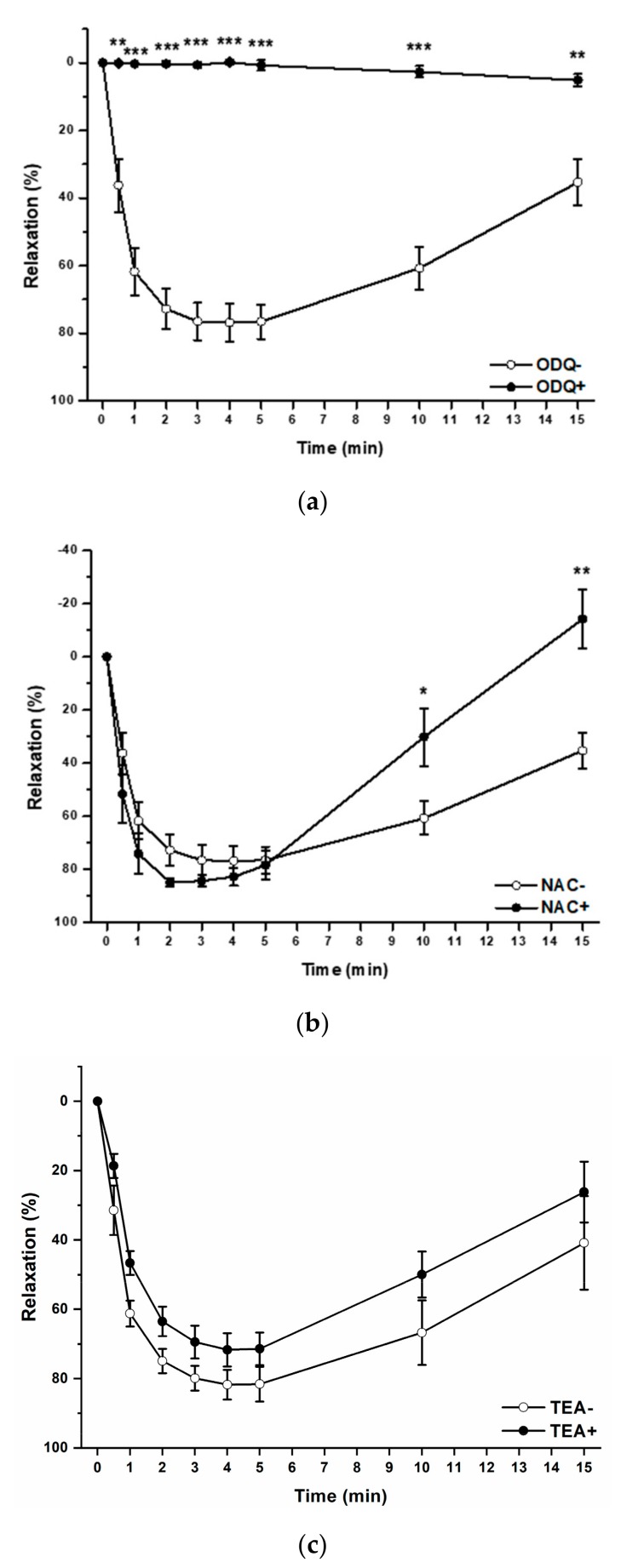
The possible mechanism of the vasorelaxation induced by H_2_S/GSNO products. The comparison of the relaxation (%) to H_2_S/GSNO products (0.5 µM) after soluble guanylyl cyclase inhibitor (ODQ; 10 µM; **a**), HNO scavenger (NAC; 1 mM; **b**) and non-specific K^+^ channel inhibitor (TEA, 1 mM; **c**). Values are mean ± S.E.M. * *p* < 0.05; ** *p* <0.01; *** *p* < 0.001 with respect to the value of the H_2_S/GSNO products response.

Next, we tried to find out the possible mechanisms of vasorelaxation induced by the H_2_S/GSNO products. We tested the involvement of the sGC signaling pathway, the nitroxyl (HNO) and the hyperpolarization in this reaction. Pretreatment with [1*H*-[1,2,4]oxadiazolo-[4,3-a]quinoxalin-1-one] (10 µM; ODQ; 20 min), an inhibitor of sGC, significantly inhibited the vasorelaxation induced by the H_2_S/GSNO products (0.5 µM) over the entire period (*n* = 8; *p* < 0.01; *p* < 0.001; Figure 5a). Incubation with an HNO scavenger, *N*-acetylcysteine (1 mM; NAC; 20 min) did not affect the maximum vasorelaxant effect induced by the H_2_S/GSNO products (*n* = 8; Figure 5b). However, NAC incubation changed the character of the response, and influenced the return of vasorelaxation (at the 10^th^ and 15^th^ min) when an inhibition was observed (*p* < 0.05; *p* < 0.01). To demonstrate the possible effect of hyperpolarization in the vasorelaxation induced by the H_2_S/GSNO products, the aortic rings were incubated with a nonspecific K^+^ channel inhibitor, tetraethylammonium chloride (1 mM; TEA; 20 min). TEA incubation had no effect on H_2_S/GSNO product-induced vasorelaxation (Figure 5c) assuming that hyperpolarization did not belong to the main vasorelaxant mechanisms of vasorelaxation of the H_2_S/GSNO products. 

## 3. Discussion

The main findings of our study are that garlic juice enhances the relaxant effect of exogenous NO donor, GSNO. To the best of our knowledge, we report for the first time that the vasoactive effect of garlic is most likely not evoked by H_2_S directly but rather by the intermediate reaction products of the H_2_S and NO interaction.

The GC-MS analysis showed the presence of sulfur-containing species in the garlic juice, which are assumed to be representatives of garlic bioactive molecules [24]. We need to keep in mind that disruption of garlic cloves leads to the activation of enzymes responsible for the formation of thiosulfinates, among which allicin is considered to be the most important biologically active compound, but it is unstable, and a bunch of degradation compounds forms during garlic processing and handling. Thus, the major organosulfur volatiles as diallyl sulfide, diallyl disulfide and diallyl trisulfide (Figure 1) are formed by the decomposition of allicin and subsequently may play a biological role through H_2_S release with involvement of thiols [25]. 

Recent studies have revealed that garlic extract has the ability to lower blood pressure in uncontrolled hypertensives [26] and suppresses the increase of systolic blood pressure with age in spontaneously hypertensive rats [1]. Takashima et al. [23] demonstrated that aged garlic extract affects the vascular endothelium, leading to vasorelaxation through promoting NO production mediated by endothelial NO-synthase. The concentration of garlic juice used in our study had no effect on the precontracted aortic rings but altered the NO donor-induced (S-nitrosogluthatione, GSNO) vasorelaxant response. Maximum GSNO-induced vasorelaxation was significantly increased after incubation with garlic juice; moreover, the time-dependent course of the response was also different in comparison to that of relaxation induced by GSNO alone (Figure 2a–d). This result could indicate a possible interaction of some compounds in garlic juice with GSNO, potentiating the release of NO. Previously it has been demonstrated that garlic extract has high NO-releasing potency expressly depending on pH [27].

H_2_S has mostly beneficial effects during oxidative stress by reacting with reactive oxygen and nitrogen species (i.e., hydrogen peroxide (H_2_O_2_), superoxide anion radical (O_2_^•^¯), hypochlorite (HOCl), or peroxynitrite (ONOO^−^)) [28,29,30,31]. Our recent study demonstrates that H_2_S is not necessarily a typical reducing agent, such as ascorbic acid or tocopherol when employed on their own. We also found that polysulfides and products of H_2_S-NO interaction are better superoxide radical (O_2_^•^¯) scavengers than H_2_S, glutathione or vitamin E derivative, modulate DNA damage and interfere with virus replication; they induce/inhibit apoptosis and modulate the intracellular calcium concentration depending on H_2_O_2_ concentration. Thus, the products of polysulfides/NO/H_2_O_2_ interaction may be involved in free radicals signaling, thus modulating oxidative and antioxidative, alternatively cytotoxic biological processes [32].

It has been reported that H_2_S potentiates the release of NO from GSNO in a pH-dependent fashion; therefore, we could assume that H_2_S may be the compound responsible for the extent of GSNO relaxation after incubation with garlic juice [16]. Evidence of the H_2_S/GSNO interaction has also been demonstrated by several other studies [16,21,33]. Studies on adult normotensive rats suggested that NO released from GSNO may interact with H_2_S to form an unknown complex directly in arterial tissue because preincubation of the rat thoracic aorta with a low concentration of H_2_S subsequently led to heightened GSNO-induced relaxation even after elimination of H_2_S from the organ bath. The authors assumed that sulfur may create component(s) in the wall of the aortic rings to induce NO release from GSNO [15,34]. We simultaneously incubated the aortic ring with an H_2_S donor to determine whether H_2_S mimics the heightened vasorelaxation recorded after incubation with the garlic juice. We incubated precontracted aortic rings with an H_2_S donor, Na_2_S, alone and followed how it affected GSNO-induced relaxation. Although incubation with H_2_S increased the maximal achieved relaxation after application of GSNO, the time-dependent characteristic of the response was not altered (Figure 3a–d). According to our results, it seems that the crucial compound in garlic responsible for extending GSNO-induced relaxation is probably not H_2_S itself. These findings are supported by our previous results reporting that the time dependence of NO release initiated by fresh garlic extract was significantly different from the time dependence of NO release evoked by Na_2_S [27]. However, we could not exclude the production of H_2_S as an intermediate reaction product during the chemical reaction with fresh garlic extract. Based on the ability of fresh garlic extract to release NO, it has been suggested at least two possible compounds that could release NO from NO donors. As H_2_S donors, organosulfur compounds in garlic extract release H_2_S via a relatively slow mechanism that requires the cooperation of endogenous thiols, such as glutathione [25,35]. The authors also indicated that H_2_S was produced by compounds that include sulfane sulfur, such as persulfides and polysulfides, or by S°-producing compounds, such as alkyl-cysteine disulfides.

These results suggest that H_2_S released from organosulfur compounds in garlic extract likely interacts with NO from GSNO, which changes its vasoactive properties. We have previously confirmed that a mixture of both donors relaxes the phenylephrine-precontracted rings of the thoracic aorta and the mesenteric artery in normotensive rats with a more than twofold potency compared with GSNO alone. Additionally, the onset of vasorelaxation of the reaction products was 7-10 times faster compared with that induced by GSNO [21]. In the present study, we also showed that the mixture of GSNO-Na_2_S products evoked a relaxation of the thoracic aorta of Wistar rats. The time-dependent curve of this reaction was very similar to that of GSNO-induced relaxation after incubation with garlic extract; nevertheless, the curve was significantly higher and faster compared to the effect induced by GSNO alone (Figure 4a–c). Several compounds have been suggested to play a role in mediating the interaction between S-nitrosothiols (RSNO) and H_2_S, altering their bioactive effects: nitroxyl (HNO), thionitrous acid (HSNO), nitrosopersulfide (SSNO^-^), N-nitrosohydroxylamine-N-sulfonate (SULFI/NO) and polysulfides (HSx^-^) [17,18,19,20,21,36]. Moreover, Cacanyiova et al. [33] studied the renal arteries of spontaneously hypertensive rats and found that the production of specific reaction products was coupled with a unique interaction between GSNO and H_2_S, as using a different chemical NO donor (DEA NONOate) in the mixture with an H_2_S donor behaved differently than application of GSNO. Cortese-Krott et al. [16] proposed SSNO^-^ to be a major longer-lived product of the sulfide/RSNOs reaction. These authors demonstrated that under conditions of excess sulfide, SSNO^-^ forms, leading to a higher rate of NO release, which activated sGC more effectively than the starting RSNO by itself. On the other hand, Filipovic et al. [18] and Wedmann et al. [37] considered HNO from sulfide/RSNOs reaction products to be the most extensive bioactive effector. They suggested that although SSNO^-^ can be produced during the sulfide/RSNOs reaction, their importance is as a generator of HNO. Similarly, Nava et al. [38] suggested that the cross-talk observed between H_2_S and NO could be mediated by HSNO and manifested as HNO.

To test the possible signaling pathways involved in the vasorelaxation of H_2_S/GSNO reaction products, we used an sGC inhibitor (ODQ), a non-specific potassium-channel inhibitor (TEA) and an HNO scavenger (NAC) (Figure 5a–c). While ODQ significantly inhibited the vasorelaxant effect, TEA had no effect on the H_2_S/GSNO products-induced relaxation. Although the pretreatment with NAC did not change the maximal vasorelaxation, the development of the vasorelaxant response over time was significantly inhibited. Thus, it seems that H_2_S/GSNO product-induced relaxation is primarily mediated by the activation of sGC and that the hyperpolarization of smooth muscle after potassium-channel activation plays no significant role in this response. H_2_S donors can evoke vasorelaxation predominantly at concentrations >100 µM, and H_2_S-induced vascular smooth muscle relaxation is mainly induced through the activation of potassium channels followed by membrane hyperpolarization [39]. Therefore, we can assume that H_2_S itself probably does not participate in the relaxation induced by H_2_S/GSNO reaction products. The involvement of the sGC signaling pathway in H_2_S/GSNO-induced relaxation has already been demonstrated. Moreover, studies have also shown that a significant portion of H_2_S/GSNO vasorelaxation is mediated by an alternative mechanism, which may directly activate sGC without releasing free NO [21]. Moreover, the inhibition of the H_2_S/GSNO products-induced vasorelaxation after NAC incubation supports the view that one of the H_2_S/GSNO products is HNO or one/more H_2_S/GSNO products are capable of releasing HNO. On the other hand, HNO has a significant effect in only the prolongation of the vasorelaxation of the H_2_S/GSNO products (the return of the reaction was faster after the HNO scavenging). According to these findings, HNO very probably does not represent the crucial vasorelaxant compound of the H_2_S/GSNO mixture. These results correlate with our previous results, when we showed that the H_2_S/GSNO products evoked similar vasoactive effect with similar mechanisms in the lobar artery isolated from hypertensive patient as well as of the renal artery isolated from spontaneously hypertensive rats [33]. 

The present study provides original data describing the vasomodulatory mechanisms of garlic juice and attempts to investigate the possible involvement of the nitroso-sulfide signaling pathway in this effect. We confirmed that organosulfur compounds of garlic juice could interact with S-nitrosothiols in a specific way, inducing enhanced vasorelaxation of the rat thoracic aorta. According to our results, we can assume that even if an H_2_S donor could potentiate NO release from a NO donor, H_2_S itself cannot be considered as a crucial bioactive compound of garlic juice. Our results indicate rather the production of new signaling molecules during the GSNO-H_2_S interaction.

## 4. Materials and Methods 

### 4.1. Guide for the Use and Care of Laboratory Animals

All experiments were performed in accordance with institutional guidelines and were approved by the State Veterinary and Food Administration of the Slovak Republic and by an ethics committee according to the European Convention for the Protection of Vertebrate Animals used for Experimental and other Scientific Purposes, Directive 2010/63/EU of the European Parliament. All rats used in this study were born in the accredited breeding establishment of the Institute of Normal and Pathological Physiology, Centre of Experimental Medicine, Slovak Academy of Sciences (INPP CEM SAS), and were housed in groups of 3 animals per cage, each strain separately, under a 12 h light-12 h dark cycle at a constant humidity (45–65%) and temperature (20–22 °C) with free access to standard laboratory rat chow and drinking water. The INPP CEM SAS provided veterinary care.

### 4.2. Chemicals

Pieces of garlic weighing 6 g were pressed through 1.2 mm diameter pores into 20 mL of buffer containing, in mM: 160 KCl, 1 MgCl_2_, 0.1 diethylenetriaminepentaacetic acid (DTPA), and 50 HEPES/TRIS, pH 7.4. The garlic homogenate was vortexed for 1 min (1800 rev/min), and the juice was extracted by separating it from debris using a Teflon glass homogenizer. Aqueous garlic extract was used fresh or was aliquoted 11 min after clove pulverization and stored at −70 °C. Frozen juice was used for several days at a concentration of 130 µM/kg homogenate. 

The stock solution of S-nitrosoglutathione (GSNO, 10 mM) was prepared by dissolving GSNO in buffer: Tris-HCl (100 mM), DTPA (0.1 mM), pH 7.4. The solution was then placed in to a −80 °C freezer until use. The concentration of the stock solution was also determined spectrophotometrically using an extinction coefficient of 922 M^−1^ cm^−1^ at 335 nm. On the day of the experiment, the stock solution (30 µL) was thawed and mixed with a buffer, Tris-HCl (200 mM); the pH was adjusted to 7.4; and the mixture was kept in sealed vials. GSNO was applied to isolated vessels at concentrations of 0.05 and 0.5 µM.

Na_2_S•9H_2_O was used as an H_2_S donor, which dissociates in water into Na^+^ and S^2−^ and reacts with H^+^ to yield HS^−^ and H_2_S. We use the term Na_2_S to encompass the total mixture of H_2_S, HS^−^ and S^2−^. The stock solution of Na_2_S (100 mM) was prepared by dissolving it in ultrapure deionized water (≥18 MΩ.cm) (Millipore, Darmstadt, Germany). The stock solution was stored in a −80 °C freezer. On the day of the experiment, the stock solution (100 µL) was thawed and mixed with a buffer, Tris-HCl (200 mM), pH 7.4. The solution was always prepared fresh before the experiment and kept in sealed vials with a minimal headspace and used immediately at concentrations of 40 µM.

The products of the Na_2_S-GSNO interaction (H_2_S/GSNO products) were generated at a 10:1 ratio (molar excess of Na_2_S over GSNO). In short, we mixed 0.5 mM GSNO with 5 mM Na_2_S at 21 ± 2 °C and waited 3 min until the reaction products were completely formed.

### 4.3. GC-MS Analysis

GC-MS analysis of garlic juice was made by Agilent Technologies 7890A with mass spectrometer (Agilent Technologies 5975C, Santa Clara, CA, USA). This instrument was operated in the electron impact (EI) mode set at electron energy 70 eV with a scan range from 33 to 330. Separation was carried out on GC-column HP-5 (Agilent Technologies, Santa Clara, CA, USA) with dimensions 30 m length × 0.25 mm diameter, and a film thickness of 0.25 μm Helium with purity 99.9995% was used as the carrier gas with a flow rate of 1.7 mL/min on the column head. The temperature of the injector was set at 280 °C. The temperature program started at 40 °C for 0 min. Subsequently, the temperature was increased with a gradient of 3 °C /min to a final temperature of 170 °C for 2 min.

The samples were prepared using liquid-liquid extraction method. 1 mL of solvent chloroform with HPLC purity was used for 2 mL of sample. After this, 1 µL of the chloroform extract was injected by autosampler (Agilent Technologies G4513A, Santa Clara, CA, USA) into the instrument with a split ratio of 20:1. 

Identification of compounds was performed by comparing the spectra with the NIST 14 library and by comparing retention times. 

### 4.4. Measurement of the Vasoactive Response and Experimental Protocols

The thoracic aorta of male Wistar rats (16 weeks old) was isolated after a brief CO_2_ anesthetization and decapitation of the animals. The 5 mm long rings were vertically fixed between two stainless wire triangles in a 20 mL incubation organ bath with Krebs solution (in mM: 118 NaCl, 5 KCl, 25 NaHCO_3_, 1.2 MgSO_4_, 1.2 KH_2_PO_4_, 2.5 CaCl_2_, 11 glucose, 0.032 CaNa_2_EDTA). The solution was oxygenated with 95% O_2_ and 5% CO_2_ and kept at 37 °C. The upper triangles were connected to isometric tension sensors (FSG-01, MDE, Budapest, Hungary), and changes in tension were registered by an AD converter NI USB-6221 (National Instruments, Austin, TX, USA and MDE, Budapest, Hungary). Changes in isometric tension were registered by the DEWEsoft (Dewetron, Prague, Czech Republic) and SPEL Advanced Kymograph (MDE, Budapest, Hungary) software. A resting tension of 1 g was applied to each ring and maintained throughout a 45 to 60 min equilibration period until stress relaxation no longer occurred.

Briefly, to test the effect of garlic juice on GSNO-induced (0.05 µM) relaxation, noradrenaline-precontracted (1 µM) aortic rings were incubated with garlic juice for 3 min. The effect of the H_2_S donor (Na_2_S) was evaluated in a similar way: after 3 min, Na_2_S pretreatment GSNO (0.5 µM) was applied. Then, the mixture of Na_2_S and GSNO (5 µM Na_2_S + 0.5 µM GSNO; see also above) was applied on the noradrenaline-precontracted rings. To confirm the involvement of the sGC signaling pathway in the relaxation effect of the H_2_S/GSNO products, an sGC inhibitor, 1*H*-[1,2,4]oxadiazolo[4,3-*a*]quinoxalin-1-one (ODQ, 10 µM), was applied 20 min before the addition of the contractile agonist. To test the assumption that the vasorelaxation of the mixture was mediated by the release of nitroxyl (HNO), experiments were also carried out after 20 min of pretreatment with an HNO scavenger, *N*-acetylcysteine (NAC, 1 mM). A nonspecific K^+^ channel inhibitor, tetraethylammonium chloride (TEA, 1 mM), was pretreated for 20 min in an organ bath to evaluate the participation of K^+^ channels in the vasorelaxation of the H_2_S/GSNO products.

First, the absolute maximal and time-dependent vasorelaxant effects of all the compounds were expressed as a percentage of the noradrenaline-induced precontraction. Second, the relative time-dependent vasorelaxant effect (the speed of vasorelaxation) of all the compounds was expressed as a percentage of the maximal relaxation. The values of the arterial tone were assessed at the 0.5^th^, 1^st^, 2^nd^, 3rd, 4^th^, 5^th^, 10^th^ and 15^th^ min after drug application.

### 4.5. Statistical Analysis

For the statistical evaluation of differences between groups, one-way analysis of variance (ANOVA) was used, followed by the Bonferroni post-hoc test. The maximum of the enriched vasorelaxation was evaluated using paired *t*-test. Differences between means were considered significant at *p* < 0.05.

## 5. Conclusions

The present study provides original data describing the vasomodulatory mechanisms of garlic juice and attempts to investigate the possible involvement of the nitroso-sulfide signaling pathway in this effect. We confirmed that organosulfur compounds of garlic juice could interact with *S*-nitrosothiols in a specific way, inducing enhanced vasorelaxation of the rat thoracic aorta. According to our results, we can assume that even if an H_2_S donor could potentiate NO release from a NO donor, H_2_S itself cannot be considered as a crucial bioactive compound of garlic juice. Our results indicate the possible production of new signaling molecules during the GSNO-H_2_S interaction.
